# Atrial Fibrillation and Ventricular Tachycardia in a Patient with Cardiac Sarcoidosis

**DOI:** 10.19102/icrm.2018.090203

**Published:** 2018-02-15

**Authors:** Joanne Lau, Huzaefah J. Syed, Kenneth A. Ellenbogen, Jordana Kron

**Affiliations:** ^1^Virginia Commonwealth University School of Medicine, Richmond, VA, USA; ^2^Division of Rheumatology, Allergy and Immunology, Virginia Commonwealth University School of Medicine, Richmond, VA, USA; ^3^Division of Cardiology, Virginia Commonwealth University Pauley Heart Center, Richmond, VA, USA

**Keywords:** Atrial fibrillation, cardiac sarcoidosis, ventricular tachycardia

## Abstract

Cardiac sarcoidosis (CS) can cause atrial and ventricular arrhythmias, conduction system disease, and congestive heart failure. The use of advanced imaging modalities including cardiac magnetic resonance and positron emission tomography with 2-deoxy-2-[fluorine-18]fluoro-D-glucose can be helpful in evaluating the extent of disease and response to treatment. The management of CS patients can be challenging, requiring immunosuppression medications, antiarrhythmic drugs, implantable cardiac devices, and cardiac ablation procedures. We report a patient with CS initially presenting with paroxysmal atrial fibrillation who later developed polymorphic ventricular tachycardia, highlighting the complexity of diagnosis and management in patients with multisystem sarcoidosis.

## Introduction

Sarcoidosis is a multisystem, granulomatous disease of unknown etiology. Patients with cardiac sarcoidosis (CS) can develop atrial and ventricular arrhythmias, conduction system disease, and congestive heart failure. The use of advanced imaging modalities including cardiac magnetic resonance (CMR) imaging and 2-deoxy-2-[fluorine-18]fluoro-D-glucose positron emission tomography (^18^F-FDG-PET) can be helpful in evaluating the extent of the disease and the patient’s response to treatment. CS patients often require management via immunosuppression medications, antiarrhythmic drugs, and implantable cardiac devices. We report a patient with CS initially presenting with paroxysmal atrial fibrillation (AF) who later developed polymorphic ventricular tachycardia, highlighting the complexity of diagnosis and management in patients with multisystem sarcoidosis.

## Case presentation

A 55-year-old Caucasian male presented to an outside clinic with a two-month history of fatigue and right bundle branch block (RBBB). At the time of presentation, he exhibited AF with a rapid ventricular response. Symptoms included shortness of breath and chest discomfort during episodes of AF, which lasted 15 to 120 minutes. Cardiac computed tomography (CT) angiography revealed a lesion in the left anterior descending coronary artery, but subsequent cardiac catheterization revealed no evidence of obstructive coronary artery disease. Notably, mildly enlarged lymph nodes in the pericardial region and mediastinum were observed on the CT angiogram. Three years prior, his left ventricular ejection fraction (LVEF) was 55% on cardiac catheterization. Two weeks prior to referral, transthoracic echocardiogram revealed an LVEF of 40%, and the newly depressed LVEF was suspected to be due to tachycardia-induced cardiomyopathy related with AF. The patient was initially started on rivaroxaban and propafenone by the referring physician. He continued to have episodes of AF and was switched to amiodarone. He was referred to our center for AF ablation.

His past medical history also included hypertension and hyperlipidemia. At the time of presentation to our center, his medications included metoprolol, rivaroxaban, and amiodarone. Due to a suspected amiodarone-induced cough and the patient’s young age, amiodarone was discontinued and changed to dofetilide.

**Figure 1:**
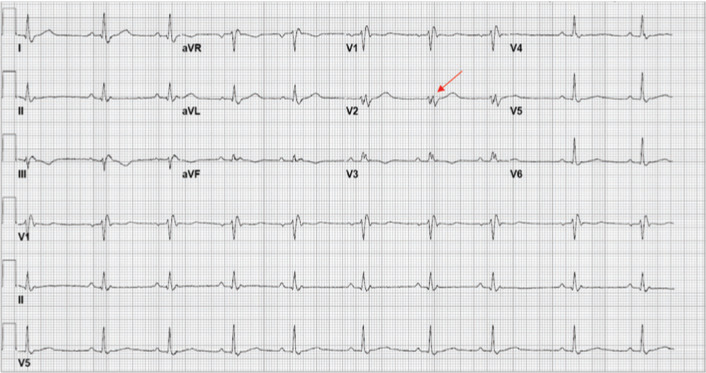
ECG showing sinus rhythm with a fractionated RBBB (arrow).

**Figure 2:**
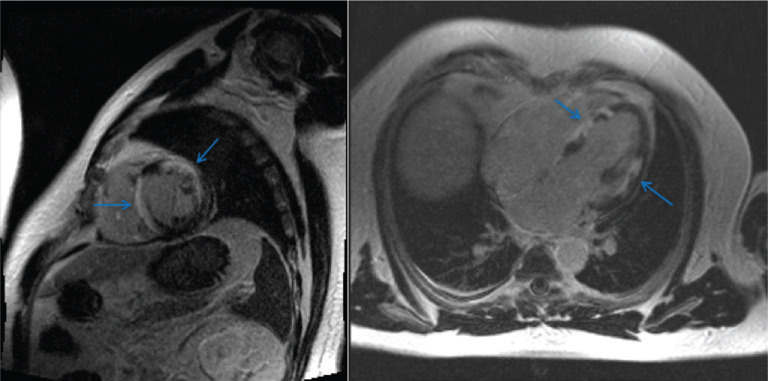
CMR imaging scan demonstrating patchy, delayed enhancement throughout the left ventricular myocardium in a nonvascular distribution pattern. Arrows indicate regions of delayed enhancement in the sagittal (left) and transaxial (right) views.

Electrocardiogram (ECG) in our clinic showed sinus rhythm with RBBB with a QRS duration of 160 ms and fragmented QRS **(Figure 1)**. He underwent cryoballoon pulmonary vein isolation (PVI) without complication. The following day, he underwent CMR to evaluate his fractionated RBBB and reduced LVEF seen on transthoracic echocardiogram and intracardiac echocardiogram during the ablation. CMR showed an LVEF of 40% and extensive, patchy delayed enhancement throughout the left ventricular myocardium along with enlarged mediastinal lymph nodes, consistent with cardiac and pulmonary sarcoidosis **(Figure 2)**.

**Figure 3:**
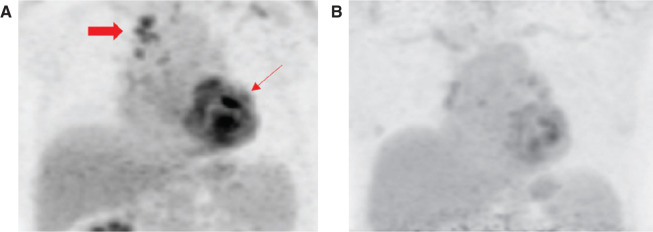
Cardiac ^18^F-FDG-PET scan showing myocardial 18F-FDG uptake. **A:** Extensive abnormal heterogeneous ^18^F-FDG myocardial uptake (thin arrow) and uptake of mediastinal lymph nodes (wide arrow). **B:** Overall decreased ^18^F-FDG uptake 10 months after the initial procedure following immunosuppressive therapy.

One week later, the patient underwent endobronchial ultrasound-guided mediastinal lymph node biopsy, which revealed poorly formed, non-necrotizing granulomas. Based on these findings, he met the 2014 Heart Rhythm Society Expert Consensus Statement criteria for the diagnosis of CS and subsequently underwent dual-chamber implantable cardioverter-defibrillator (ICD) implantation for primary prevention.^1^ The patient was reluctant to use corticosteroids and was instead started on methotrexate with folic acid. A baseline ^18^F-FDG-PET/CT scan showed extensive heterogeneous ^18^F-FDG uptake throughout the myocardium that corresponded with perfusion abnormalities on the myocardial perfusion imaging scan **(Figure 3A)**. However, although his shortness of breath resolved, the patient’s complaint of fatigue remained.

Based on the active inflammation seen on cardiac PET and the patient’s reduced LVEF, the methotrexate dosage was increased from 15 mg to 25 mg weekly; 400 mg infliximab was intravenously administered at baseline, weeks two and six, and then every six weeks thereafter.

**Figure 4:**
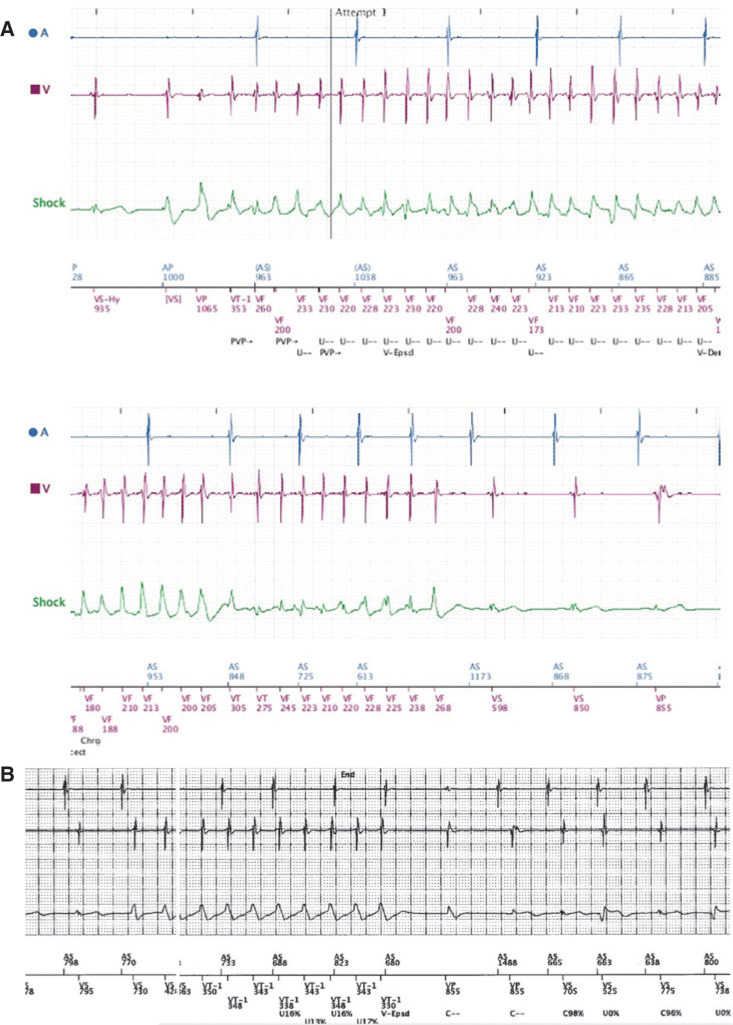
**A:** Atrial and ventricular electrograms from a dual-chamber ICD showing non-sustained polymorphic ventricular tachycardia with ventriculoatrial dissociation. Arrhythmia correlates with episode of presyncope. **B:** An example of non-sustained monomorphic ventricular tachycardia.

**Figure 5:**
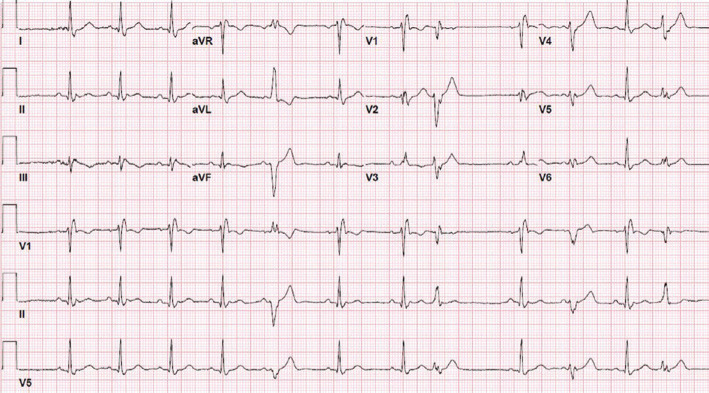
Twelve-lead ECG recorded at the time of admission for multiple presyncopal spells showing polymorphic premature ventricular contractions, normal QTc interval, and RBBB.

Seven months after cardiac ablation, the patient experienced multiple presyncopal episodes while at work. ICD remote transmission showed five episodes of non-sustained polymorphic ventricular tachycardia (VT), as well as episodes of monomorphic VT **(Figure 4)**. ECG was performed and showed normal sinus rhythm with frequent premature ventricular contractions **(Figure 5)**. The patient was admitted to the hospital for initiation of sotalol. On discharge, the frequency of infliximab treatment was increased to every four weeks. No programming changes were made to the ICD.

Transthoracic echocardiogram revealed stable LVEF of 40%. A cardiac ^18^F-FDG-PET scan performed 10 months after the initial ^18^F-FDG-PET scan revealed an overall decrease in ^18^F-FDG uptake, with a new focus in the mid-inferolateral myocardial wall **(Figure 3B)**. On the current regimen, the patient has remained clinically stable without any further atrial or ventricular arrhythmias.

## Discussion

The prevalence of sarcoidosis is estimated to be 4.7 to 64 in 100,000 people, with a higher incidence in African-American and northern European populations.^2^ Young and middle-aged African-American females are at greatest risk, followed by African-American males and Caucasian females, in order of prevalence.^3^ In one retrospective study, cardiac involvement in sarcoidosis was diagnosed more frequently in males.^4^ Although sarcoidosis is typically thought of as a disease of African-American females, a high degree of suspicion is required to diagnose CS, regardless of the presenting patient’s sex or race.

Myocardial involvement in sarcoidosis occurs in up to 25% of cases in the United States and may be the cause of 13% to 25% of deaths from the disease.^5^ Despite its high contribution to mortality, CS is often clinically silent, and only 40% to 50% of patients with CS at autopsy are correctly diagnosed with such during the patient’s lifetime.^6^

We described a male Caucasian patient with CS who presented with AF. The precise incidence of atrial arrhythmias in CS patients remains unknown. AF can be the first presenting manifestation.^7^ In one cohort of patients with CS, 32% were reported to have supraventricular arrhythmias with the following types: AF, 18%; atrial tachycardia, 7%; atrial flutter, 5%; and atrioventricular nodal reentrant tachycardia, 2%.^8^ Atrial arrhythmias in CS are common but occur less frequently than ventricular arrhythmias.^9^ Mechanisms of atrial arrhythmias in CS include triggered activity, abnormal automaticity, and reentry.

Granulomas on necropsy in CS patients have been found as histologic evidence of the disease in the left ventricular free wall, basal ventricular septum, right ventricle, papillary muscles, and right and left atria, in the order of decreasing frequency.^10^ Although atrial dilation secondary to left ventricular dysfunction is known to contribute to the onset of atrial arrhythmias, atrial granulomas may also lead to scarring and atrial arrhythmias such as AF.^10^ Delayed gadolinium enhancement technology exists to visualize atrial scarring on CMR, but to our knowledge, the use of atrial delayed enhancement has not been studied in CS patients and should be a focus of future research.^11^

There are limited data on whether catheter-based PVI is effective in CS patients with AF. In a prior case in which AF was the initial presentation of CS, the patient had recurrent AF after PVI but experienced a decreased AF burden following immunosuppressive treatment.^12^ In another study of nine CS patients with atrial arrhythmias, two patients with paroxysmal AF and three with persistent AF underwent PVI; one individual with persistent AF later experienced reccurence.^13^ Our patient had no recurrence of AF on ICD interrogation following cryoballoon PVI, although immunosuppression was initiated several months after ablation on the basis of extensive ^18^F-FDG uptake on PET. Evidence supporting the use of immunosuppression to treat atrial arrhythmias in sarcoidosis is limited to case reports.^12,14^ Many experts state that it would be useful to test the utility of immunosuppression to treat AF in patients with CS.^1^

Despite presenting with AF and having a moderately depressed LVEF at diagnosis, our patient subsequently developed poly- and monomorphic VT. The 2008 American College of Cardiology/American Heart Association/Heart Rhythm Society (HRS)’s device-based guidelines support the performance of ICD implantation in patients with CS, regardless of left ventricular function, as a class IIA recommendation (level of evidence: C).^15^ CS patients with preserved or mildly reduced left ventricular function may still be at risk for ventricular arrhythmias.^16^ The 2014 HRS expert consensus statement attempted to further risk stratify CS patients in terms of sudden cardiac death (SCD) based on reduced right or LVEF, indication for pacemaker implantation, unexplained syncope or near-syncope, or late gadolinium enhancement on CMR.^1^ The present case illustrates that early diagnosis of CS and SCD risk stratification, with ICD implantation when appropriate, are critical in this patient population. A dual-chamber ICD was implanted in this particular case because the patient had significant sinus bradycardia and RBBB. The capacity to pace the atrium allowed for b-blocker titration to treat the cardiomyopathy, and for antiarrhythmic drug titration as needed to treat AF and/ or other arrhythmias, should they occur. On follow-up device interrogation, the patient was paced in the atrium up to 60% of the time, suggesting an atrial lead was needed to provide an adequate chronotropic response.

The risk of stroke in CS patients with AF is not known. From the time of diagnosis, our patient was anticoagulated with a novel oral anticoagulant based on his CHA_2_DS_2_-VASc score. Although no data exist on the risk of thromboembolism in sarcoidosis patients with AF or on the effect of anticoagulation in this group, CS patients with AF should receive thromboprophylaxis in accordance with guidelines for non-valvular AF.^1^ Patients with sarcoidosis are at higher risk for pulmonary embolism, raising the concern that systemic inflammation may increase the risk of hypercoagulability in this population.^17^ Medications such as b-blockers, calcium channel blockers, sotalol, dofetilide, and amiodarone can also be used in the course of treatment, though there are no comparative data to guide antiarrhythmic drug selection.^1^ Sotalol is often a safe and effective choice in patients with CS as it treats both atrial and ventricular arrhythmias and avoids the risks of pulmonary and hepatic toxicities that can occur with amiodarone. This is advantageous, as many patients may have concomitant pulmonary and hepatic sarcoidosis.

## Conclusions

We presented the case of a 55-year-old male with paroxysmal AF and fractionated RBBB who was diagnosed with CS. Despite having only a moderately depressed LVEF, the patient subsequently developed ventricular arrhythmias. This complex case illustrates several critical teaching points about CS: (1) regardless of a patient’s sex or race, a high index of suspicion is needed to diagnose CS; (2) the risk of SCD should be considered, and an ICD implanted when indicated; and (3) the management of these patients, which may include the use of immunosuppression medication, antiarrhythmic therapy, cardiac device implantation, and cardiac ablation, is challenging and requires ongoing vigilance. Further research should be conducted to improve the handling of these individuals.
